# Cascade recurring deep networks for audible range prediction

**DOI:** 10.1186/s12911-017-0452-2

**Published:** 2017-05-18

**Authors:** Yonghyun Nam, Oak-Sung Choo, Yu-Ri Lee, Yun-Hoon Choung, Hyunjung Shin

**Affiliations:** 1 Department of Industrial Engineering, Ajou University, Suwon, Korea; 2 Department of Otolaryngology, Ajou University School of Medicine, Suwon, Korea

**Keywords:** Hearing Aids, Hearing improvement, Neural networks, Deep learning, Cascade structure, Recurrent structure

## Abstract

**Background:**

Hearing Aids amplify sounds at certain frequencies to help patients, who have hearing loss, to improve the quality of life. Variables affecting hearing improvement include the characteristics of the patients’ hearing loss, the characteristics of the hearing aids, and the characteristics of the frequencies. Although the two former characteristics have been studied, there are only limited studies predicting hearing gain, after wearing Hearing Aids, with utilizing all three characteristics. Therefore, we propose a new machine learning algorithm that can present the degree of hearing improvement expected from the wearing of hearing aids.

**Methods:**

The proposed algorithm consists of cascade structure, recurrent structure and deep network structure. For cascade structure, it reflects correlations between frequency bands. For recurrent structure, output variables in one particular network of frequency bands are reused as input variables for other networks. Furthermore, it is of deep network structure with many hidden layers. We denote such networks as cascade recurring deep network where training consists of two phases; cascade phase and tuning phase.

**Results:**

When applied to medical records of 2,182 patients treated for hearing loss, the proposed algorithm reduced the error rate by 58% from the other neural networks.

**Conclusions:**

The proposed algorithm is a novel algorithm that can be utilized for signal or sequential data. Clinically, the proposed algorithm can serve as a medical assistance tool that fulfill the patients’ satisfaction.

## Background

As individuals have longer life expectancies, the quality of life has become more and more important nowadays as well as auditory rehabilitation of hearing impaired persons. In the recent years, the need of hearing aid(HA)s for patients with hearing loss is spreading widely however, the satisfaction levels of HAs are quite diverse among individuals. One of the many causes that affect the satisfaction levels is the composite interactions between the variables that affect the outcomes of HAs. Walden et al [1] investigated the correlations between the patients’ demographic information and hearing test results in 50 patients that were successfully using HAs, and determined that age is a major variable for successful HA use. In a study of acute hearing loss, 83 patients were classified into four levels in a range of 1 to 4 according to their degrees of recovery. The factors that affected the hearing recovery were analyzed using nonparametric statistical analysis methods. The results indicated that the presence of tinnitus and/or dizziness, duration of hearing loss, pure tone audiometry patterns, degree of hearing loss, and age have statistically significant effects on recovery [2]. Although other studies have also reported the reasons for HA failure and methods to improve HA outcomes, studies with large number of patients for HAs have not been investigated.

For successful use of HAs, a highly reliable prediction model attributing to specific characteristics of HAs and frequencies is essential. Although there are algorithms for fitting of HAs developed by various HA manufacturing companies, objective information on the degree of hearing improvement obtained by different HAs in patients are sometimes inaccurate or concealed. Mulrow et al. [3] proposed a logistic regression model for variables such as age, education, functional limitations, and the degree of hearing loss using data from 176 patients but this model showed low accuracy of training data and testing data (75–88% and 54–84%, respectively). Cvorovic et al. [4] presented a multiple linear regression model with 541 Swiss patients that showed sudden sensorineural hearing loss symptoms as a diagnosis model [4]. However, this model has limitations as it was limited to sudden sensorineural hearing loss, the validity of the model was not verified, and its applicability in the clinical field was not evaluated.

**Fig. 1:**
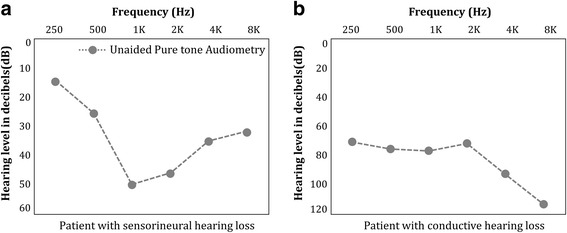
Audiometry of patients with different category and type of hearing loss. **a**. Patient with sensorineural hearing loss. **b**. Patient with conductive hearing loss

Figure 1 shows the unaided pure tone audiometry of two patients with different category and type of hearing loss. In Fig. 1a, the patient has sensorineural and convex type hearing loss. Sensorineural hearing loss occurs due to abnormalities in the cochlea or disorders in the nerves that connect the inner ear with brain. In addition, due to convex type, the patient has hearing loss in medium frequency bands (1KHz, 2KHz). The threshold of medium frequencies is higher than that of low frequencies and high frequencies by at least 15 dB. Thus, the patient is unable to hear sounds such as cell phone bell. Figure 1b shows the audiometry for patient with conductive hearing loss. For this patient, all sound thresholds from low frequency bands to high frequency bands exceed 65 dB and the degree of hearing loss is severe. In both cases, a model that can reflect patient information, clinical evaluations, and hearing aid information is required for HA fitting suitable for each patient. If the hearing gain by HAs can be predicted individually after HA fitting, it will be possible to adjust and regulate the hearing gain sequentially. This will then enable the use of HA to reach to its optimal hearing gain resulting in high satisfaction levels.

Therefore, the purpose of this study was to develop a new model that can present the expected degrees of hearing gain followed by HA fitting based on the variables that can affect the outcomes of HAs. This model is expected to fulfill the patients’ expectation levels, motivate the patients to use and manage HAs, and help to maximize the hearing improvement through application of HAs in the clinical fields.

## Neural networks general

The proposed algorithm is based on neural networks. Neural networks are machine learning algorithms used for prediction or classification. It is known to show high prediction performance even in cases where the relationships between input variables and target variables have not been defined or are complicated [5]. Out of various neural networks the one that is most frequently used is the Multilayer Feedforward Network which is composed of three layers; input layer, hidden layer, and output layer [6]. Each of the nodes in a neural network are connected in the forward direction, from the input layer to hidden layer and from hidden layer to output layer. With selection of appropriate numbers of the hidden nodes and output nodes, it is known to show high prediction performance [5, 7, 8]. Given the structure of a neural network, the learning process begins with the given dataset. For *n* instances, ***x*** = {*x*_*i*_ | *x*_*i*_ ∈ *R* ^*d*^, *i* = 1, 2, …, *n*} and *k* target variables, ***y*** = {*y*_*j*_ | *y*_*j*_ ∈ *R*, *j* = 1, 2, …, *k*}, the learning process of a neural network is to estimate the weight ***w*** that connects each nodes. The estimation of ***w*** is calculated in the direction of reducing the error, which is the difference between output variable ***f*** = {*f*_*j*_ |*f*_*j*_ ∈ *R*, *j* = 1, 2, …, *k*} and target variable ***y***. Thus, the objective is to minimize the sum of squared errors and the optimization problem is:

$$ \min \kern0.5em {\left(\boldsymbol{f}-\boldsymbol{y}\right)}^T\left(\boldsymbol{f}-\boldsymbol{y}\right). $$

In this study, we propose a novel neural network that have three structural characteristics: cascade, recurrent, and deep network structure. Previous studies concerning each of the three structures are reviewed as the following.

### Cascade structure

When a neural network obtains high prediction performance, selecting an appropriate number of hidden nodes is important. Although the number of hidden nodes is selected by trial-and-error, in general, there has been extensive studies in determining the number of hidden nodes through algorithm. One of the representative algorithms is a cascade-correlation neural network which selects the optimal number of hidden nodes by adding a single hidden node at each step in the training process. In addition, it achieves faster training process than general neural networks [9, 10].

### Recurrent structure

Neural networks in recurrent structures are frequently utilized for the prediction of time series or sequential data. For instance, in predicting *k* target variables, ***y*** = [*y*_1_, *y*_2_, … *y*_*k*_], that has characteristics of time-series, the output variables are correlated with each other. In such case, higher performance can be obtained by utilizing previous step *y*_*t*-1_ as an input variable for prediction of ***t***-time step *y*_*t*_. This is well consistent with the concept of Recurrent Neural Network that combines a general neural network with the notion of time series. In recurrent neural network, hidden nodes are utilized as storages that preserve the information in previous training step [11, 12].

### Deep network structure

In Recent works, it has shown that piling up many layers in a neural network leads to improvements in prediction performance. The basic concept of deep neural networks is to pile up many hidden layers between the input layer and the output layer [13]. However, if a neural network becomes deep with many layers, it leads to difficulty in learning weights and thus the overfitting problem [5]. To solve the overfitting problem, the Restricted Boltzmann Machine and the Deep Belief Network can be used. The Restricted Boltzmann Machine is a model made by removing connections between layers from Boltzmann machine and updates the entire parameters by piling up hidden layers one by one [14]. The Deep Belief Network is a model that piles up pre-trained layers with unsupervised learning [15].

In this study, we proposed a novel neural network algorithm by incorporating advantages of three neural network structures described above.

## Methods

**Fig. 2:**
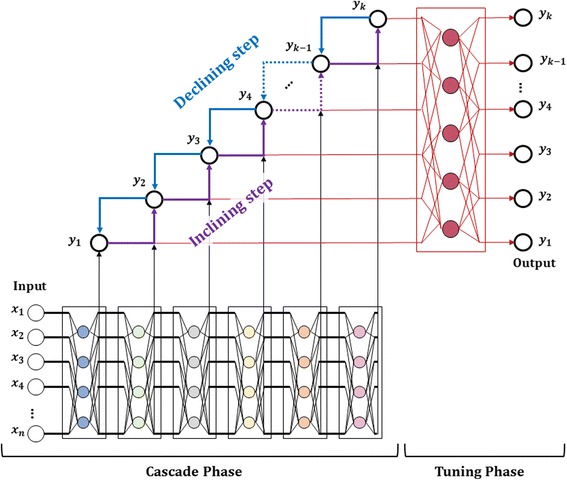
Cascade Recurring Deep Network: The proposed algorithm consists of two phases; Cascade Phase and Tuning Phase

The proposed algorithm is a type of neural network that can be applied to signal data in which output variables are closely correlated with each other. In the case of neural networks with many output variables, learning of weight ***w*** is difficult because there are many connections to hidden nodes. On the contrary, if neural networks are independently configured for each output variable, it would be difficult to determine the number of hidden nodes. To circumvent the difficulty, we propose a neural network algorithm that reflects the structural advantages of the cascade-correlation network, recurrent neural network, and deep neural network explained above. The proposed algorithm is a Cascade Recurring Deep Network (CRDN) that is suitable for predicting signal or time series data in which output variables are closely correlated. Figure 2 shows the structure of the CRDN. Cascade structures refer to configuring neural networks in progressive order for each output variable, and recurrent structures refer to reusing one output variable as an input variable for another network. Also, deep network structures refer to piling up *k* + 1 hidden layers for *k* output variables. CRDN consists of two phases; cascade phase and tuning phase. In cascade phase, a neural network for one output variable is configured and sufficiently trained. Then the trained network is used as *base leaner* for constructing the next neural network and the process progressively carried out for other output variables. In tuning phase, the errors in the output values from cascade phase are corrected.

### Cascade phase

Cascade phase is step for training output variables in stepwise fashion. When we predict a specific output variable, other correlated output variable is utilized as an input variable. It is known that for training neural networks increase in the number of nodes leads to higher computational cost and difficulty in training the weight ***w*** [5]. Therefore, in cascade phase, for predicting many output variables, we first construct a feedforward neural network for one output variable and the trained network is used as *base leaner* for constructing the another neural network. The base learner is updated as the output variables are changed. Such processes have an advantage of drastically reducing the training time by reusing previously trained feedforward neural networks. Cascade phase progresses in bi-directional order; inclining step, which is a learning process in the forward direction, and declining step, which is a learning process in the reverse direction.

#### Inclining step

Inclining step is a process where output variables are learned progressively. When we have *k* target variables, ***Y*** = {***y***_***j***_ | ***y***_***j***_ ∈ *R* ^*m*^, *j* = 1, 2, …, *k*}, and *k* output variables, ***F*** = {***f***_***j***_ |***f***_***j***_ ∈ *R* ^*m*^, *j* = 1, 2, …, *k*}, we can construct a neural network to predict **y**_**j**_. There may be several dimensions of each target variable, but we only consider case of one dimension (m = 1) to easily describe the problem. For inclining step, firstly we construct a neural network *net*_1_ that predicts ***y***_**1**_. Output ***f***_**1**_ is obtained by [*f*_1_, ***w*** ^(1)^] = *net*_1_(***w*** ^(0)^, ***x*** ^(1)^, *y*_1_) where ***w*** ^(**1**)^ is trained weight matrix of neural network for predicting ***y***_**1**_, ***w*** ^(**0**)^ is randomly configured weight matrix for initialization, and ***x*** ^(1)^ = [*x*_1_, *x*_2_, …, *x*_*d*_] is input vector for predicting ***y***_**1**_. When the neural network *net*_1_ has been sufficiently trained, we construct neural network *net*_2_ that predicts ***y***_**2**_. For training *net*_2_, the output value ***f***_**1**_ is added to input vector ***x***. Since the two target variables **y**_**1**_ and ***y***_**2**_ affect each other, weight matrix ***w*** ^(**1**)^ that has been previously trained is utilized as a *base learner*. Then, output value ***f***_**2**_ is obtained by [***f***_2_, ***w*** ^(2)^] = *net*_2_(***w*** ^(1)^, ***x*** ^(2)^, ***y***_2_) where **w** ^(**2**)^ is trained weight matrix for predicting **y**_**2**_, ***x*** ^(2)^ = [*x*_1_, *x*_2_, …, *x*_*d*_, ***f***_1_] is input vector for predicting **y**_**2**_. Then, neural networks for the remaining output variable are learned in the same manner. In general, the final predicted value ***f***_***k***_ is calculated by

$$ \left[{\boldsymbol{f}}_{\boldsymbol{k}},\ {\boldsymbol{w}}^{\left(\boldsymbol{k}\right)}\right] = n e{t}_k\left({\boldsymbol{w}}^{\left(\boldsymbol{k}-1\right)},\ {\boldsymbol{x}}^{\left(\boldsymbol{k}\right)},\ {\boldsymbol{y}}_{\boldsymbol{k}}\right) $$

where ***w*** ^(***k***)^ is resulting trained weight matrix for net_k_, ***x*** ^(***k***)^ = [x_1_, *x*_2_, …, *x*_*d*_, ***f***_***k*** − 1_] is input vector for *net*_*k*_.

#### Declining step

When all neural networks have been learned through the inclining step, learning in the reverse direction is carried out in the same manner.

$$ \left[{\boldsymbol{f}}_{\boldsymbol{k}-1},\ {\boldsymbol{w}}^{\left(\boldsymbol{k}-1\right)}\right]= n e{t}_{k-1}\left({\boldsymbol{w}}^{\left(\boldsymbol{k}\right)},\ {\boldsymbol{x}}^{\left(\boldsymbol{k}-1\right)},\ {\boldsymbol{y}}_{\boldsymbol{k}}\right) $$

where **w** ^(**k**)^ is trained weight matrix in final inclining step, ***x*** ^(***k*** − 1)^ = [*x*_1_, *x*_2_, …, *x*_*d*_, ***f***_***k***_] is input vector. When the declining step has been carried out for all output variables, the cascading phase is completed. The final output values ***f*** ^***A***^ = [*f*_1_, *f*_2_, …, *f*_*k*_] reflect the correlations between all adjacent variables.

### Tuning phase

Tuning phase is the step for correcting the errors in the final output values from cascade phase by constructing a error-correction network. The final output values ***F*** ^**A**^ = [***f***_1_, ***f***_2_, …, ***f***_***k***_] in cascade phase are input nodes and target variables ***Y*** = [***y***_1_, ***y***_2_, …, ***y***_***k***_] are output nodes for the error-correction network. The constructed network is a type of auto associative neural network in which input nodes and output nodes are similar to each other. The necessity of tuning phase can be justified as following. In cascade phase, the output variable *f*_*k*_ of *k* ^th^ neural network *net*_*k*_ is utilized as an input variable of the (*k* + 1)^th^ neural network. Since every output variable accompany errors the input variable ***f***_***k***_ contains errors that must be corrected. It is known that error-correction networks can improve prediction performance in deep neural networks [16, 17].

## Experiments

### Data

A total 2,182 patients that were diagnosed with hearing loss and treated with hearing aids at the Department of Otolaryngology, Ajou University Hospital, Suwon, Republic of Korea between January 2001 and February 2016 were enrolled in the study. Among them, 45 patients who were either younger than 2 years old, totally deaf (100 dB or higher), or had no pure tone audiometry(PTA) data with or without HAs were excluded from the study. Following the approval from the Institutional Review Boards of the Ajou University School of Medicine, a retrospective chart review was done, and all possible factors related to hearing or outcomes of HAs were collected. The analysis factors were divided into three categories; patient information, clinical evaluation, and HA information as shown in Table 1. From the patient information category, age, sex, duration of hearing loss, and presence of tinnitus or dizziness were used as variables. Also, pure tone hearing threshold without HAs, degree of hearing loss, type of hearing loss, and word recognition scores(WRS) were used as variables in the clinical evaluation category. As for variables of HA, duration of hearing impairment without HAs, site of HAs (left, right, or both), program design of HAs(digital, analogue, programmable), types of HAs (behind the ear(BTE) type, in the ear(ITE) type, in the canal (ITC) type, completely in the canal(CIC) type) were analyzed. Types of hearing loss were divided into five groups; ascending type, descending type, flat type, concave type, and convex type using low (0.25, 0.5 kHz), mid (1, 2 kHz), and high frequency (4, 8 kHz) thresholds in PTA before using HAs. The ascending type was defined in cases where the threshold of low frequencies was at least 15 dB higher than the average threshold of high frequencies, and the descending type was defined in cases where the threshold of high frequencies was at least 15 dB higher than the threshold of low frequencies. In cases of flat type hearing loss, differences between the thresholds of all frequencies were smaller than 15 dB. The concave type hearing loss was diagnosed when the threshold of medium frequencies was at least 15 dB lower than that of low and high frequencies. Lastly, in the convex type, the hearing thresholds of medium frequencies were at least 15 dB higher than that of low and high frequencies. The degrees of hearing loss without wearing HAs were classified into mild (25 ~ 39 dB), moderate (40 ~ 59 dB), high (60 ~ 79 dB), and severe (80 ~ 100 dB) degrees based on the hearing thresholds (average dB of 0.5,1,2, and 3 kHz) in PTA.

**Table 1:** Data Description

Input Variables	
Patient Information	Age, Sex, Underlying Diseases, Experience of Hearing Aids, Side of Hearing Aids
Clinical Evaluations	Unaided Pure Tone Audiometry, Unaided Hearing in noise test, Threshold per frequency, Category of hearing loss, Degree of hearing loss, Type of hearing loss, Tinnitus status, Average air conduction hearing threshold, Average bone conduction hearing threshold, Mean word recognition score
Hearing Aid(HA) Information	Models of HA, Number of channels, Types of Has, Tinnitus treatment option, Frequency Transposition, Type of microphone, Ventilation, Feedback cancellation
Target Variables	
Hearing gain	PTA after wearing HAs (250Hz, 500Hz, 1KHz, 2KHz, 4KHz, 8KHz)

To check the hearing gain, PTA and WRS tests were done before and after 8 to 12 weeks of HA fitting, which were compared and analyzed. These results were used as output variables of the proposed algorithm. The PTAs were done using hearing thresholds from 0.25 to 8 KHz, and the average of hearing thresholds of 0.5,1,2, and 3 kHz were calculated. For WRS, patients were provided with test word sounds at their most comfortable listening level and instructed to repeat or write down the words and numbers that were accurately heard and understood. These results were then converted into percentages as WRS. Although the most comfortable listening levels before and after using HAs were not the same in some cases, the WRS were still compared due to the fact that they were values under the most comfortable threshold condition.

### Correlation in target variables

**Fig. 3:**
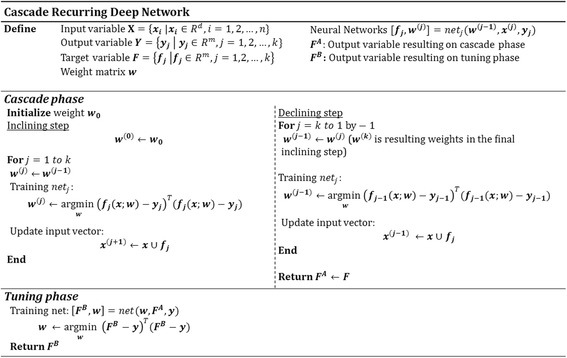
Pseudo code for Cascade Recurring Deep Network

**Fig. 4:**
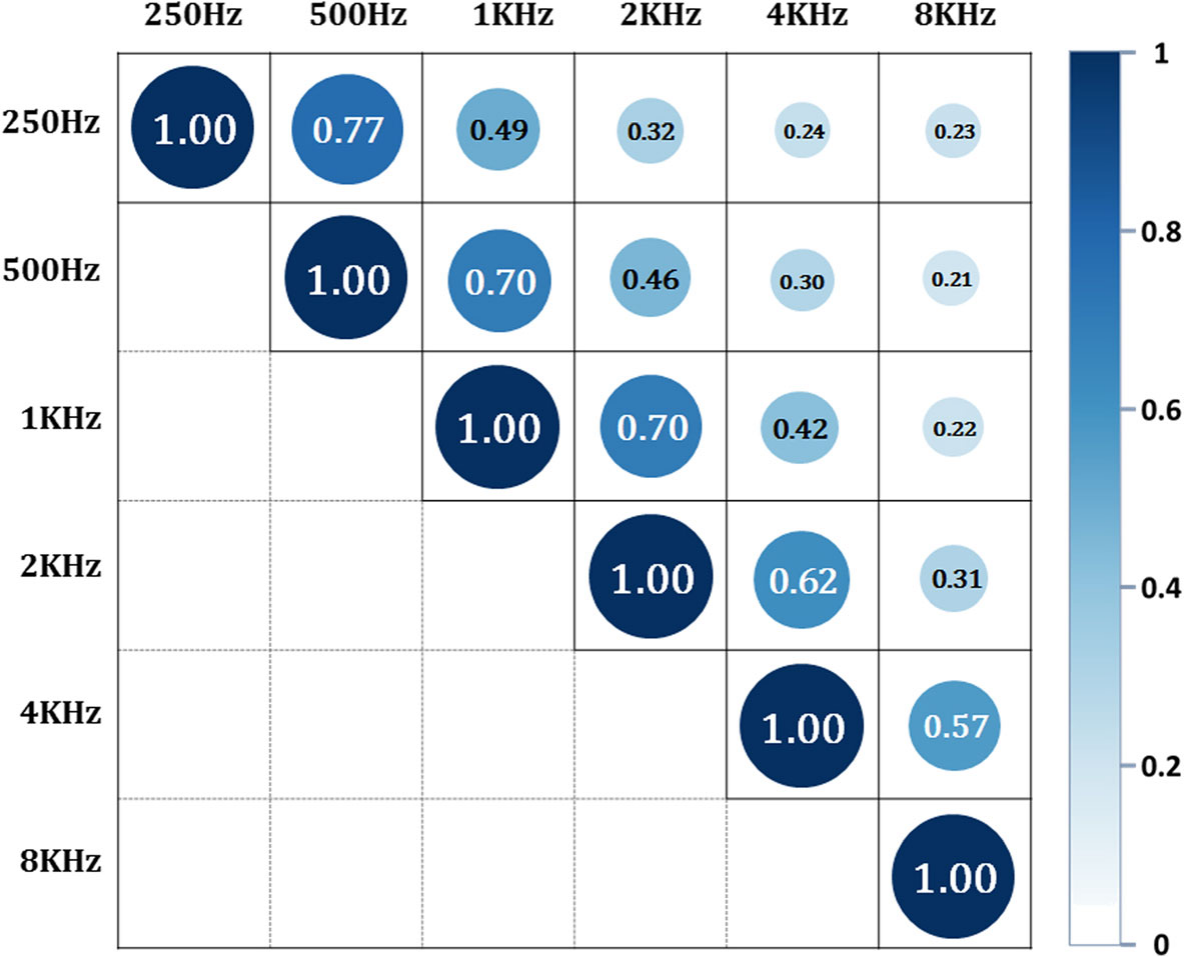
Pearson correlation coefficients for target variables

To apply the proposed algorithm, we checked existence of correlation between target variables which are hearing gains. Figure 4 shows the Pearson correlation coefficients between the target variables. From the Fig. 3, it shows that low frequency bands, 250Hz and 500Hz have a high correlation with 0.77. Also, we can see that two adjacent frequency bands show correlations of at least 0.57 (from 4 KHz and 8 KHz). For two bands, 250Hz and 8 KHz, which are far away from each other, it shows a positive correlation of 0.23 indicating that all frequencies bands are correlated with each other. Therefore, we can infer that adjacent frequencies are highly correlated and that the all frequency bands have positive correlations.

### Experimental settings

**Fig. 5:**
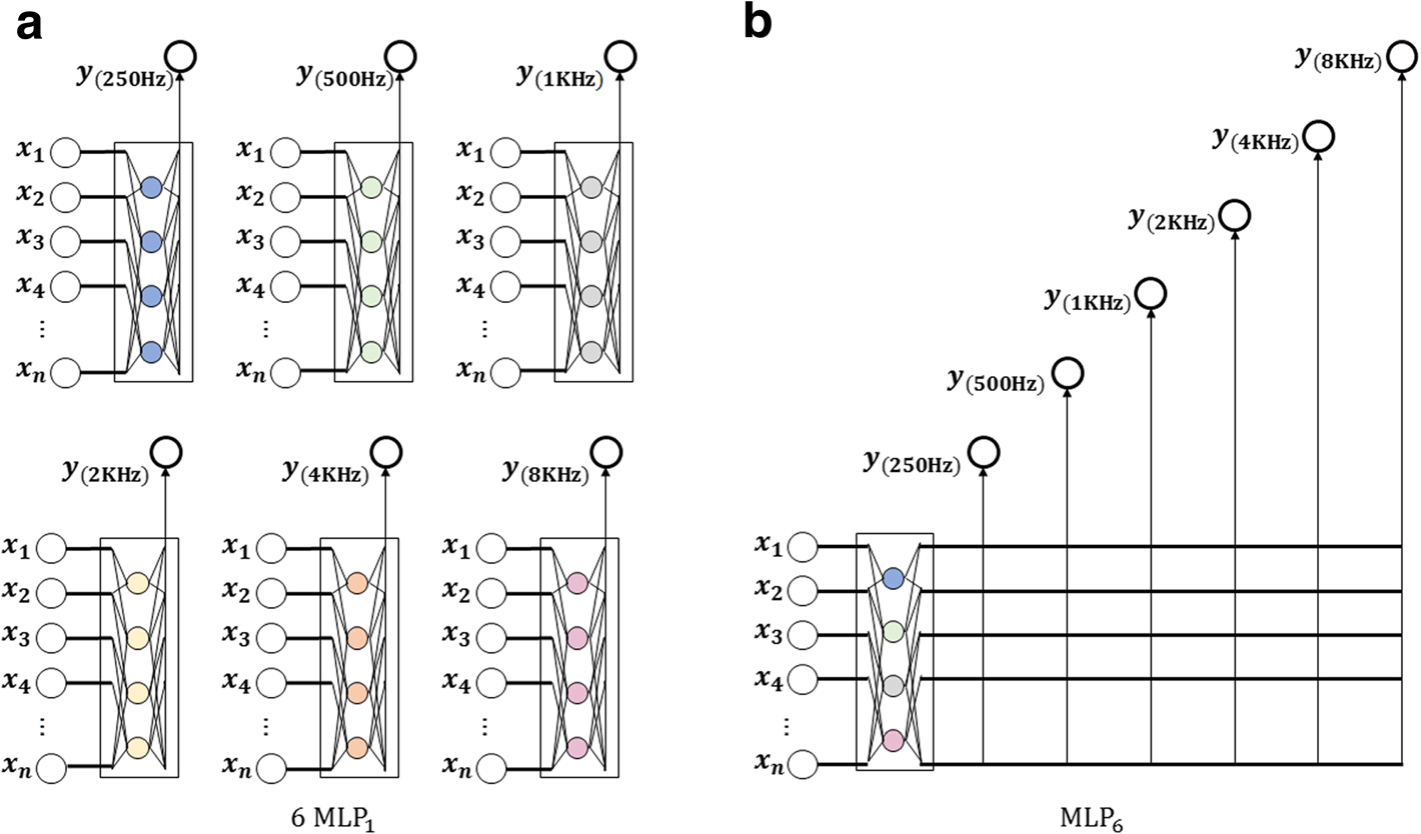
Comparison model: **a** 6MLP_1_s: MLP with a single output node, **b** MLP_6_: a single MLP with 6 output nodes

To compare the prediction performance of the proposed algorithm that reflect correlations between frequencies, a neural network in the structure as shown in Fig. 5 was designed. To predict frequencies, neural network solves a regression problem. The structure of neural network *net*_*j*_ is a three layered Perceptron structure ([Input Layer]-[Hidden Layer]-[Output Layer]) having 31 input nodes, 20 ~ 30 hidden nodes on average, and one output node [7]. In other words, it has Multi-Layer Perceptron structure of [Input: 31]-[Hidden: (20 ~ 30)]-[Output: 1]. Cascade Recurring Deep Network has the structure described in Fig. 2. In the inclining step of the cascading phase, the neural network is learned in the direction from the low frequency (250 Hz) to the high frequency (8 KHz). In the declining step, the neural network is learned in the reverse direction from the high frequency (8 KHz) to the low frequency (250Hz). For instance, in the first inclining step, neural network *net*_250*Hz*_ for predicting frequency band of 250Hz is sufficiently trained. Then, network *net*_500*Hz*_ for predicting frequency band of 500Hz utilizes *net*_250*Hz*_ as a *base learner*. For training net_500Hz_, the output variable *f*_250*Hz*_ of net_250Hz_ is utilized as an input variable and the weight ***w*** ^(250*Hz*)^, which has been sufficiently trained, is utilized as the initial weight for *net*_500*Hz*_. The process progressively carried out up to frequency band of 8KHz. In the declining step, the final network *net*_8KHz_ in the inclining step is used as a base learner, and similar process to inclining step is carried out in reverse direction from frequency band of 8KHz down to 250Hz. In the tuning phase, neural networks are trained by setting the final outputs in cascade phase *f*_250*Hz*_, *f*_500*Hz*_, …, *f*_6*KHz*_ as input node and target variables *y*_250*Hz*_, *y*_500*Hz*_, …, *y*_6*KHZ*_ as output nodes. Through the cascading phase and tuning phase, we can construct a neural network that incorporates all the effects of other frequency bands on one particular frequency band. Fig. 4a shows neural networks, MLP_1_, that predict each of six frequency bands, where the networks are independent Multi-Layered Perceptron for one output. Figure 5b shows neural network, MLP_6_, that predicts 6 frequency bands at once. Six MLP_1_s and MLP_6_ only consider patient information and clinical evaluations as input variables. For comparison of prediction performances, the optimal number of hidden node was selected in between 20 to 30 for each networks. The percentages of randomly sampled training, validation, and test sets were 40, 30, and 30%, respectively. The whole experiments were repeated 10 times. The prediction performance was measured with Mean Absolute Percentage Error (MAPE) where smaller values imply higher prediction performance [18]. MAPE is expressed in percentages and is given as the following:

$$ \mathrm{MAPE}=\frac{1}{n}{\displaystyle \sum_{i=1}^n}\left|\frac{y_i-{f}_i}{y_i}\right|\times 100 $$

where, n is the number of entire data, *y*_*i*_ is target values, *f*_*i*_ is output values.

## Results and discussion

### Results for validity of CRDN

**Fig. 6:**
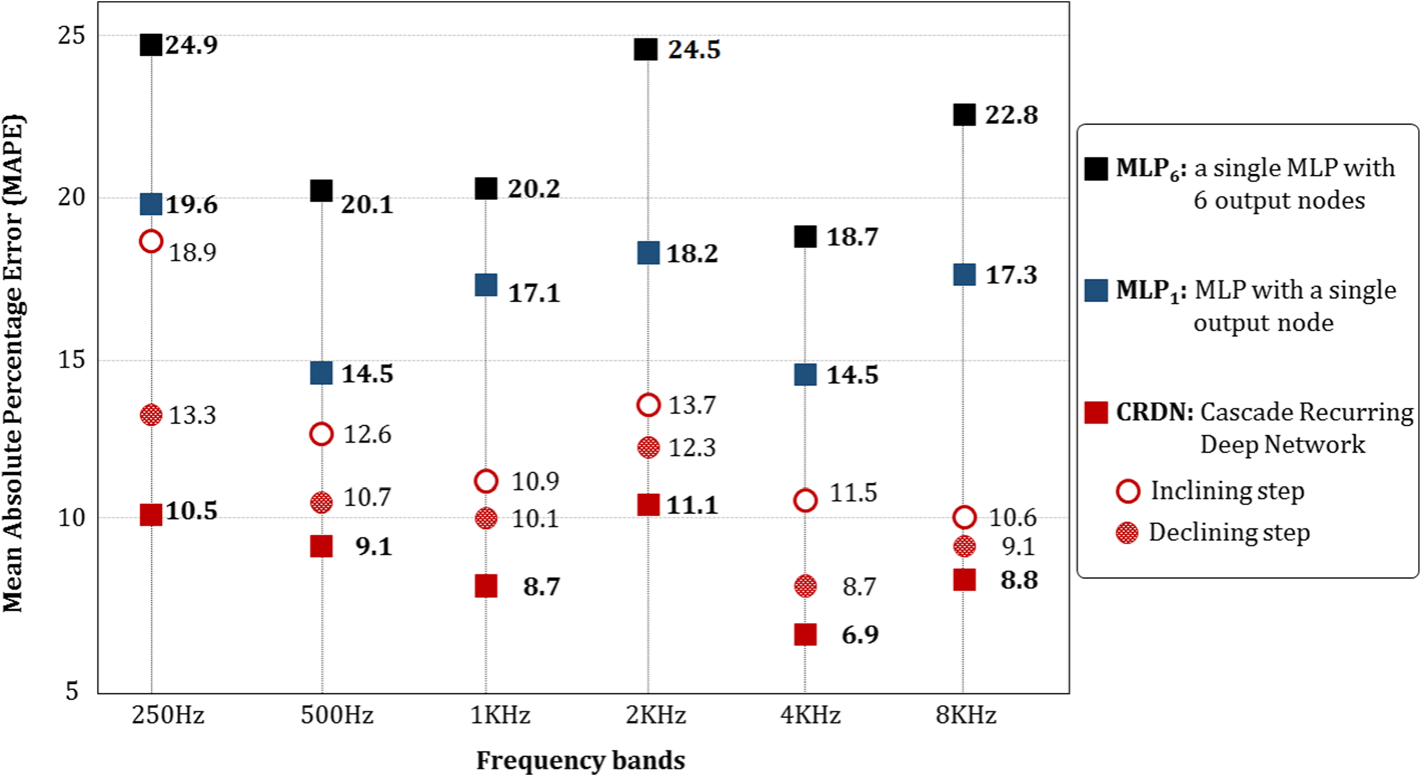
Error comparison: When we compare the proposed algorithm with other algorithms (6MLP_1_, and MLP_6_), CRDN showed lower mean error rate of 9.2% for six frequency bands (250Hz, 500Hz, 1KHz, 2KHz, 4KH, 8KHz)

Figure 6 shows comparison of MAPE of three types of neural networks (CRDN, 6MLP_1_, and MLP_6_) by frequency bands. CRDN shows the lowest error rates in all frequency bands (Avg. 9.2%). On the contrary, six MLP_1_s and MLP_6_ showed error rates of 21.8 and 16.8% on average respectively. We could deduce that CRDN, which reflects every correlation between target variables, shows highest performance, followed by MLP_6_, which can reflect some proportion of correlations between target variables. In addition, the error rates of inclining and declining in CRDN are 13.1 and 10.7% on average respectively. If we compare the error rate of CRDN (Avg. 9.2%) that had been trained up to the last step, tuning phase, we could deduce that considering adjacent frequencies more often leads to improvement in performance.

The low error rate of CRDN has meaningful implications in clinical aspects. Since hearing tests only select representative frequency bands, it is difficult to measure the actual thresholds in PTA of adjacent frequency bands. It only approximates the thresholds with median of measured PTA of adjacent frequency bands. For instance, a 4 K-dip from noise-induced hearing loss not only has high thresholds in PTA for 4KHz but also affects adjacent frequency bands. Since CRDN structure considers such influence, it is bound for successful results.

### Results for utility of CRDN

**Fig. 7:**
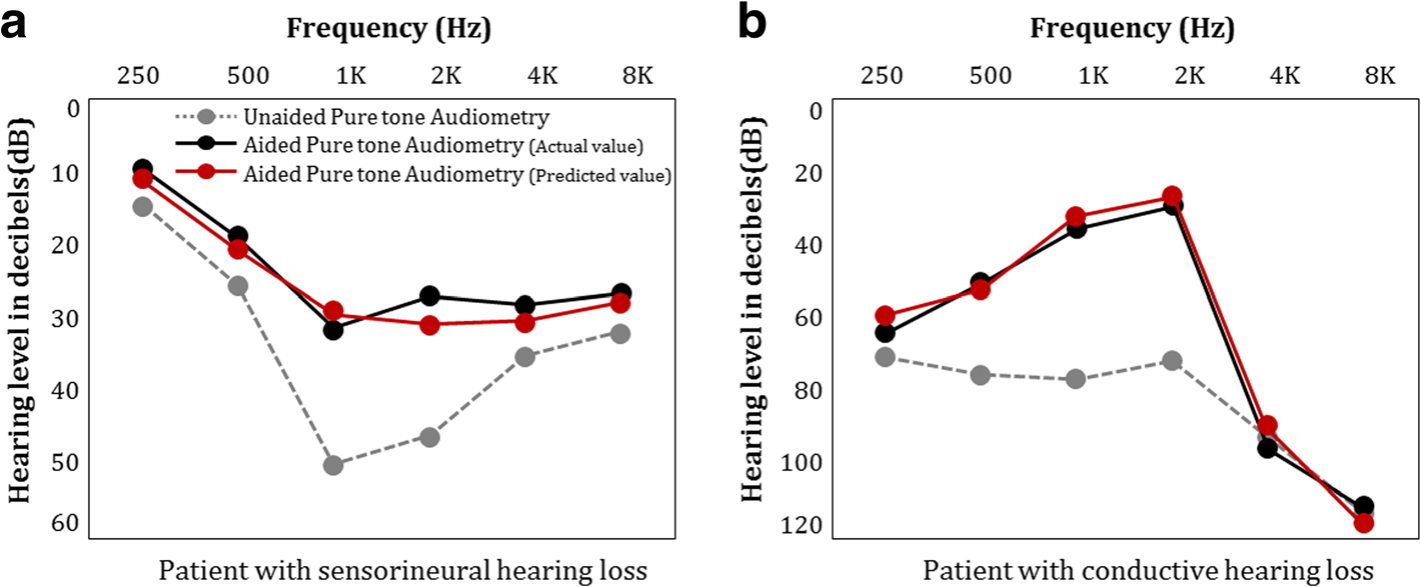
Comparison result after application of CRDN. **a**. Patient with sensorineural hearing loss. **b**. Patient with conductive hearing loss

To verify the utility of CRDN, the proposed algorithm was applied to actual patients. We show cases of patients with sensorineural hearing loss and patients with conductive hearing loss. Figure 7a and b represent the results of hearing gain with CRDN for each case.

#### Case I: patients with sensorineural hearing loss

Figure 7a shows a case of a patient with sensorineural hearing loss with convex type who have hearing loss in frequency bands (1KHz, 2KHz). Sensorineural hearing loss occurs due to abnormalities in the cochlea or disorders in the nerves that connect the inner ear with the brain.

For convex type, the threshold of medium frequencies is higher than that of low frequencies and high frequencies by at least 15 dB. In such cases, the patients with hearing loss cannot hear the sounds of daily conversations. To resolve the hearing loss, we applied CRDN to patients with sensorineural hearing loss. The expected degrees of hearing gains with CRDN was fitted with actual hearing gain, and the results show high satisfaction levels.

#### Case II: patients with conductive hearing loss

Figure 7b shows a case of a patient with conductive hearing loss. Conductive hearing loss results from damage to the path for delivery of sounds from the external ear to the middle ear. In the case of this patient, all sound thresholds from low frequency bands to high frequency bands exceed 65 dB and the degree of hearing loss is severe. Since the hearing thresholds for daily conversations are 20 ~ 70 dB, the patient has difficulties in daily life. The CRDN provided outstanding hearing gains by amplifying the frequencies to appropriate levels.

Although the patients have different characteristics of hearing loss, patient information and clinical evaluations for both cases, CRDN can provide suitable hearing gains for individual patients. On the basis of results, CRDN can serve as a medical assistance tool that fulfill the patients’ satisfaction levels, and helps to maximize the hearing improvement through application of HAs in the clinical fields.

## Conclusion

In this study, we propose a novel neural network algorithm that provides expected degrees of hearing gain for patients with hearing loss who wear hearing aids. The proposed algorithm is a Cascade Recurring Deep Network that reflects correlations between adjacent frequencies. This is a deep network that piles up same number of hidden layers as that of target variables such that it can be utilized for signal or sequential data. Also, it takes the structural advantages of cascade-correlation networks and recurrent neural networks.

In algorithmic perspective, CRDN has novelty in following aspects. CRDN has a scalable structure that can be applied to various signal or sequential data, and achieves faster training time since it progressively piles up the layers. CRDN reuses neural networks that previously have been trained and it not only achieves fast training time but also reflects the correlations between output variables. In addition, since CRDN uses weights that previously have been learned, it can reduce the time of learning weights in predicting new output variables. In the experiments, the mean error rate of CRDN in six frequency bands (250Hz, 500Hz, 1KHz, 2KHz, 4KHz, 8KHz) was 9.2%. When compared to other neural network models that does not reflect correlations between frequencies, it showed that the mean error rate can be reduced by 58%. In this paper, we only compared performance of CRDN with two types of MLP. As an extension of the comparison, we can consider other machine learning algorithms such as support vector machine, regularized regression analysis, etc.

In clinical perspective, since CRDN provides more accurate information on expected hearing gain after wearing hearing aids, it can reduce the gap between expected and actual experience of wearing hearing aids. Therefore, it can serve as an effective medical assistance tool. Further works include applying the proposed algorithm to other types of signal or sequential data.
